# Improved Liver R2* Mapping by Averaging Decay Curves

**DOI:** 10.1038/s41598-017-05683-5

**Published:** 2017-07-21

**Authors:** Xinyuan Zhang, Jie Peng, Changqing Wang, Yanqiu Feng, Qianjin Feng, Xinzhong Li, Wufan Chen, Taigang He

**Affiliations:** 1 Guangdong Provincial Key Laborary of Medical Image Processing, School of Biomedical Engineering, Southern Medical University, Guangzhou, China; 2 School of Automation Engineering, University of Electronic Science and Technology of China, Chengdu, China; 3 Schools of Medicine & Dentistry, Plymouth University, Plymouth, UK; 4 Cardiovascular Sciences Research Centre, St George’s University of London, London, UK; 5 Royal Brompton Hospital and Imperial College, London, UK

## Abstract

Liver R2* mapping is often degraded by the low signal-to-noise ratio (SNR) especially in the presence of severe iron. This study aims to improve liver R2* mapping at low SNRs by averaging decay curves before the process of curve-fitting. Independently filtering echo images by nonlocal means (NLM) demonstrated improved quality of R2* mapping, but may introduce new errors due to the nonlinear nature of the NLM filter, during which the averaging weights may vary with different image contents at multiple echo times. In addition, the image denoising effect of the NLM may decline when no sufficient similar patches are available. To overcome these drawbacks, we proposed to filter decay curves instead of images. In this novel scheme, decay curves were averaged in a local window, each with a weight assigned according to the curve-similarity measured by the distance between one of the neighboring curves and the targeted one. The proposed method was tested on simulated, phantom and patient data. The results demonstrate that the proposed method can provide more accurate R2* mapping compared with the NLM algorithm, and hence has the potential to improve diagnosis and therapy in patients with liver iron.

## Introduction

The thalassemia major, a common genetic blood disease, poses a threat to human health worldwide. For the patients with β thalassemia major, the intestinal boosting absorption of iron because of the anemia and the repeated blood transfusion lead to tissue with iron overload which can produce toxicity and cause complications. The liver is the major site of iron storage and the liver iron concentration (LIC) has been used as a surrogate measure of total body iron stores^1^. Accurate and robust measurement of LIC is thus of primary importance in clinical practice for guiding and monitoring the therapy of the thalassemia major while avoiding the adverse effect of excess chelator administration^2, 3^.

A variety of methods have been presented for the quantification of LIC, including serum biochemical test, hepatic needle biopsy and magnetic resonance imaging (MRI)^2^. The MRI R2 and R2* relaxation rates have gained worldwide acceptance in the diagnosis and monitoring the therapy of iron-overloaded thalassemia^4–7^. It has been reported that the relaxometry decay rates R2 and R2* (the reciprocal of T2 and T2* relaxation times) have a good correlation with the biopsy-proven LIC^6, 8–10^ and can be converted to the LIC by established calibration curves^11, 12^.

For the R2* measurement, multiple echo images at different echo times (TEs) are generally acquired. A region-of-interest (ROI) is firstly delineated in the homogenous parenchyma area without vessels and then the averaged signal intensity of all pixels in the ROI at each echo image is fitted to generate the representative R2* for the LIC quantification^7, 13^. The ROI approach has an advantage of avoiding contributions from vascular and biliary structures and is thus accurate and popular. However, the iron in the tissue may be uneven. By providing only an averaged liver R2* value, the ROI method is unable to assess iron distribution across the liver. Alternatively, the pixel-wise fitting approach produces a R2* mapping which has the potential to reflect the iron distribution over the entire liver slice and may contain clinically relevant information for advanced tissue characterization. Unfortunately, the pixel-wise fitting may suffer from the low signal-to-noise ratio (SNR) problem, especially in heavily iron-overload livers, and result in inaccurate R2* measurement^14^.

To reduce the noise impact on R2* mapping, the combination of a nonlocal means (NLM) filter and a noise corrected curve-fitting method^15^ has been proposed for accurate R2* mapping even in the presence of severe iron overload^16, 17^. The NLM filter^18^ calculates the weighted mean intensity of pixels in an image by taking advantage of the redundancy of image structure. In order to preserve edges while denoising, the averaging weights in the NLM algorithm are adapted to the local content of each individual image. Thus, the filtering using the NLM algorithm is a nonlinear process. It should be noted that each echo image was filtered individually in the previous report^15^. In this scenario, the nonlinear nature of the NLM filter may introduce distortions into decay curves due to different image contrast with varying TEs. The distortion of decay curves will consequently degrade the accuracy of R2* mapping. In addition, the noise suppression performance of the NLM filter degrades if no sufficient similar patches are found.

To avoid potential distortion of the decay curve due to nonlinear filtering, we propose to filter pixel-wise decay curves instead of multiple TE images. In this novel scheme, the decay curve of each pixel is considered a basic unit to be processed. The decay curves similar to the targeted curve in a relatively large search window are identified and assigned high weights in the averaging process. In this way, the tiny structural details are expected to be preserved better than those processed by the NLM filter. In this study, the proposed filtering algorithm which Averages Decay Signals with Similarity-based Weights was abbreviated as the ADSSW algorithm. To evaluate its performance, the ADSSW was compared with the NLM algorithm on simulated, phantom and *in vivo* data.

## Materials and Methods

### The NLM and ADSSW algorithms

The multi-echo R2* images often need to be pre-preprocessed to reduce the noise impact on decay curves. However, there has been limited data in this research field. Our pioneering work using the well-known NLM filter proved to improve the performance of R2* mapping significantly compared with the conventional Gaussian filter^15^. In this study, therefore, the ADSSW algorithm was compared to the NLM algorithm only to avoid redundancy. The ADSSW and NLM algorithms will be described in the following subsections.

#### The NLM algorithm

Let ${\boldsymbol{m}}({x})={{m}}^{{k}}({x}),{k}\in {\mathcal{K}}{,}{\mathcal{K}}=\{1,2,\ldots {K}\}$ indicate the serial images acquired at different TEs. *K* is the total number of TEs. *x* denotes the spatial position of the pixels from one of *K* images. The NLM algorithm filters each echo image individually by calculating the weighted average intensity of pixels in a search window, which can be formulated as^18^:

1 $$\overline{{{m}}^{{k}}({{x}}_{{i}})}=\sum _{{{x}}_{{j}}\in {{V}}_{{i}}}{{w}}^{{k}}({{x}}_{{i}}{,}{{x}}_{{j}}){{m}}^{{k}}({{x}}_{{j}}),{k}\in {\mathcal{K}}$$

where *x*_*i*_ denotes the position of current pixel to be filtered, *V*_*i*_ the neighbourhood of the pixel *x*_*i*_, *m* ^*k*^(*x*_*j*_) the intensity of the pixel *x*_*j*_ in the *k*th image, and *w* ^*k*^(*x*_*i*_, *x*_*j*_) the weight between the two pixels *x*_*j*_ and *x*_*i*_ in the *k*th image. $\overline{{{m}}^{{k}}({{x}}_{{i}})}$. a weighted average, is the output at pixel *x*_*i*_ for the *k*th image.

To calculate the filtered intensity of the pixel *x*_*i*_, those pixels having similar local patterns to that of the pixel *x*_*i*_ are assigned large weights. The weight can be formulated as:

2 $${{w}}^{{k}}({{x}}_{{i}}{,}{{x}}_{{j}})=\exp (-{{G}}_{{a}}{\Vert {{m}}^{{k}}({{N}}_{{{x}}_{{i}}})-{{m}}^{{k}}({{N}}_{{{x}}_{{j}}})\Vert }^{2}/{h})/{{z}}_{{i}}^{{k}},\forall {{x}}_{{i}}\ne {{x}}_{{j}}$$

Herein, ||·|| denotes the Euclidean distance. ${{N}}_{{{x}}_{{i}}}$ and ${{N}}_{{{x}}_{{j}}}$ denotes the small neighborhood of the pixel *x*_*i*_ and *x*_*j*_, respectively. *G*_*a*_ is a normalized Gaussian function (the SD of *a*) that gives more weight to pixels near the centre. Parameter *h* is the decay rate of weights and controls the degree of smoothing and usually determined by *h* = *βσ*, where *β* is scalar and *σ* is the SD of the noise in the image. ${{z}}_{{i}}^{{k}}$ is the normalizing factor: ${{z}}_{{i}}^{{k}}=\sum _{{{x}}_{{j}}\in {{V}}_{{i}}}{{w}}^{{k}}({{x}}_{{i}},{{x}}_{{j}})$. To avoid the over-weighting due to the self-similarity when *x*_*j*_ = *x*_*i*_, the weight *w* ^*k*^(*x*_*i*_, *x*_*i*_) is calculated as:

3 $${{w}}^{{k}}({{x}}_{{i}}{,}{{x}}_{{i}})=\,{\max }\{{{w}}^{{k}}({{x}}_{{i}}{,}{{x}}_{{j}}),\forall {{x}}_{{j}}\ne {{x}}_{{i}}\}$$

The NLM algorithm keeps details in an image by assigning large weights to those pixels with similar local patterns according to their intensity distance *d* = $\parallel {{m}}^{{k}}({{N}}_{{{x}}_{{i}}})-{{m}}^{{k}}({{N}}_{{{x}}_{{j}}}){\parallel }^{2}$ as in Eq. (2). The smaller the distance *d*, the more similar the neighborhood patches become. However, if applied directly to each of the TE images individually, the weight of the same spatial position *x*_*j*_ at different TE images may be different due to the varying image intensity with the decay. Thus, the NLM algorithm, a nonlinear filter in nature, may introduce additional errors to the decay curves. Another limitation of the NLM algorithm is that its denoising performance depends on the number of available similar patches for average. In the extreme case that the central patch cannot find a similar one in the search window, high-contrast details may be blurred in the filtered output^19^.

#### The ADSSW algorithm

As mentioned, pixel-wise curves are averaged in the proposed algorithm:

4 $$\overline{{\boldsymbol{m}}({{x}}_{{i}})}=\sum _{{{x}}_{{j}}\in {{V}}_{{i}}}{w}({{x}}_{{i}}{,}{{x}}_{{j}}){\boldsymbol{m}}({{x}}_{{j}})$$

where ***m***(*x*_*j*_) is a vector of size *K* indicating the decay signals at *x*_*j*_, *K* is the total number of TEs, *w*(*x*_*i*_, *x*_*j*_) is the weight between ***m***(*x*_*j*_) and ***m***(*x*_*i*_), $\overline{{\boldsymbol{m}}({{x}}_{{i}})}$ is a weighted mean vector, the output signal at pixel *x*_*i*_. Unlike the NLM algorithm, the weight *w*(*x*_*i*_, *x*_*j*_) is calculated from the distance between two signal vectors at *x*_*i*_ and *x*_*j*_ not two patches

5 $${w}({{x}}_{{i}}{,}{{x}}_{{j}})=\exp (-||{\boldsymbol{m}}({{x}}_{{i}})-{\boldsymbol{m}}({{x}}_{{j}})|{|}^{2}/{h})/{{z}}_{{i}}^{{k}},\forall {{x}}_{{i}}\ne {{x}}_{{j}}$$

6 $${w}({{x}}_{{i}}{,}{{x}}_{{i}})=\,{\max }\{{w}({{x}}_{{i}}{,}{{x}}_{{j}})\},{{x}}_{{j}}\ne {{x}}_{{i}}$$

In the proposed ADSSW algorithm, if the decay signals are close to each other, the distance between the decay curves would be small and the weight between them would be large. Similar to the NLM algorithm, the weight of the current decay curve was set to the maximum of the weights of its surrounding decay signals (Eq. [6]) to avoid the impact of the self-similarity.

### Curve-fitting method

The performance of the R2* measurement is affected by curve-fitting models^13^. To improve the accuracy and precision of the liver R2* measurement, the commonly used curve-fitting models include the offset model^20^, the truncation model^21, 22^, the first-moment and the second-moment noise-corrected model (M^1^NCM and M^2^NCM)^16, 23^. It has been demonstrated that the offset model often overestimates the R2* values and its accuracy decreases with the decrease of SNR. Although the truncation model is superior to the offset model, it underestimates the very high R2* value especially at a low SNR. By contrast, the M^1^NCM and M^2^NCM models consistently produce accurate and precise R2* values across all R2* values and SNR levels^16, 23–26^. Although advantageous over the M^1^NCM in terms of simple form and fast fitting, the M^2^NCM model produces a slightly higher SD than the M^1^NCM model. Thus, we adopted the M^1^NCM model in our study to fit the signal intensities at all TE images for the liver R2* measurement:

7 $$E({S}_{M})=\sigma \sqrt{\frac{\pi }{2}}\frac{(2L-1)!!}{{2}^{L-1}(L-1)!}{}_{1}F_{1}(-\frac{L}{2},L,-\frac{{S}_{{0}}{e}^{-TE\cdot {R}_{2}^{\ast }}}{2{{\rm{\sigma }}}^{2}})$$

where *S*_*M*_ denotes the observed signal, *E*(*S*_*M*_) the mathematical expectation of *S*_*M*_, i.e., the first moment of *S*_*M*_, σ the SD of noise, *L* the number of channels, !! the double factorial: *n*!! = *n*(*n*−2)(*n*−4)…, _1_ *F*_1_ the confluent hyper-geometric function, *TE* the echo time, *S*_0_ the noise-free signal intensity at *TE* = 0, and *R*2* the rate of the relaxation. The nonlinear Levenberg-Marquardt algorithm was implemented for curve fitting^27^ with positive constraints imposed on the parameters *S*_0_ and *R*2*. The initial values of the *S*_0_ and *R*2* were tentatively set to be the maximum signal intensity on each pixel and the inverse of the 0.5 times the maximum *TE*, respectively.

The M^1^NCM model directly fits the measured data to the first moment of *S*_*M*_, *i.e*., the expectations of the signals. The NLM and ADSSW algorithms decrease the SD of the magnitude noise so to produce improved expectations hence improved R2* mapping.

### Numerical Simulations

The true liver R2* mapping was unknown in practice. In order to validate the efficacy of our proposed algorithm, the synthetic liver R2* mapping was developed that could serve as a “gold standard”. A liver mask, which include the liver parenchyma and vessels, was developed by Feng *et al*.^24^ and used to generate ten referenced R2* mappings with fixed vessel R2* value (33 s^−1^) and varying liver parenchyma R2* values ranging from 100 s^−1^ to 1000 s^−1^ with an increment of 100 s^−1^. The different liver parenchyma R2* values from low to high represent those with LIC from normal to severe, respectively. For each reference R2* mapping, 12 noise-free echo images were synthesized using the following model:

8 $${I}^{k}(x)=\{\begin{array}{c}{S}_{0}{e}^{-TE(k)^{\ast}R{2}_{p}^{\ast}},\quad if\,x\in {{\rm{\Omega }}}_{p}\\ {S}_{0}{e}^{-TE(k)^{\ast}R{2}_{v}^{\ast}},\quad if\,x\in {{\rm{\Omega }}}_{v}\end{array}$$

where *I* ^*k*^(*x*) is the ideal image intensity at spatial position *x* and the *k*th echo time *TE*(*k*)*, S*_0_ is the intensity of the image at *TE* = 0, Ω_*p*_ and Ω_*v*_ represent the typical location of parenchyma and vessel, respectively, *R*2_p_* and *R*2_v_* denote the R2* values of the parenchyma and vessel, respectively. The values of TEs are the same as those used for *in vivo* data, *i.e*., 12 TEs of 0.93, 2.27, 3.61, 4.95, 6.29, 7.63, 8.97, 10.40, 11.8, 13.2, 14.6, and 16 ms. Subsequently, the noise-free serial images were used to generate Rician distributed serial images with three varying SNRs of 15, 30, 60. The SNR is defined as *SNR* = *S*_0_/*σ*, where *σ* is the standard deviation (SD) of noise and *S*_0_ was set to 200 in the numerical simulations.

### Phantom study

A phantom, containing 13 tubes filled with MnCl_2_ of different concentrations ranging from 0 to 24 mM, was used for improved validation. The phantom data was acquired using a multi-echo gradient-echo sequence on a 1.5 T whole-body scanner (Avanto, Siemens) with the following parameters: flip angle of 5°, repetition time of 200 ms, 16 TEs of 0.97 1.84 2.71 3.58 4.45 5.32 6.19 7.06 7.93 8.80 9.67 10.54 11.41 12.28 13.15 and 14.02 ms, slice thickness of 5.5 mm, bandwidth per pixel of 2300 Hz, matrix of 128 × 128, in-plane resolution of 3.1 × 3.1 mm^2^. To evaluate the robustness of each method (*i.e*., fitting the noisy, NLM- and ADSSW-filtered images with M^1^NCM model) on a wide spectrum of SNRs, a smaller flip angle of 5 degree is used to obtain a low SNR data for each acquisition and produce nine varying SNRs data with the average numbers of 1, 2, 4, 8, 16, 32, 64, 128, and 256.

### *In vivo* study

In this study, 128 subjects with normal to severe LIC were retrospectively selected and investigated. The varying R2* values correspond to varying LICs defined as follows^6, 8^: for normal LIC, R2* value < 158 s^−1^; for mild LIC, 158 s^−1^ < R2* < 370 s^−1^; for moderate LIC, 370 s^−1^ < R2* < 714 s^−1^, for severe LIC, R2* value > 714 s^−1^. Imaging was carried out on a 1.5 T Siemens Sonata Scanner (Siemens Medical Solutions, Erlangen, Germany). This study was approved by NRES committee London-East, 26/05/2011 and the methods were carried out in accordance with the relevant guidelines and regulations. The informed consent was obtained for experimentation with human subjects. A fat-saturated fast multi-echo gradient-echo sequence with single-slice acquisition was implemented with the following parameters: flip angle of 20°, repetition time of 200 ms, 12 TEs of 0.93, 2.27, 3.61, 4.95, 6.29, 7.63, 8.97, 10.40, 11.8, 13.2, 14.6, and 16 ms, slice thickness of 10 mm, bandwidth per pixel of 1955 Hz, matrix of 64 × 128, in-plane resolution of 3.1 × 3.1 mm^2^, and number of averages of 1. Herein, the flip angle of 20° has been demonstrated to be optimal to compromise image quality and acquisition speed (breath-hold requirement)^6, 15, 28^ and the multi-echo images were attained supine within a breath-hold of approximately 13 seconds.

### Evaluation of the R2* mapping

For the simulation study, the R2* mappings obtained from the original noisy images, the NLM-filtered, and the ADSSW-filtered data were compared. Meanwhile, the error image of the R2* mapping, which was the difference between the referenced true R2* mapping and the R2* mapping fitted from the noisy and filtered data, was also presented. In order to provide a more intuitive visual comparison, several estimated R2* values and truth R2* values in a line just across the liver parenchyma and blood vessels were plotted. The Root Mean Squared Error (RMSE) is used as a quantitative measure to evaluate the accuracy of R2* mapping:

9 $$RMSE=\sqrt{(\sum _{i}{({R}_{i}-{\hat{R}}_{i})}^{2}/M)}$$

where *R*_*i*_ and ${\hat{R}}_{i}$ are the true R2* and the estimated R2* values at pixel *i* in the R2* mapping, respectively; *M* is the total number of R2* values in the R2* mapping. A smaller RMSE means a more accurate R2* mapping. For the statistic analysis, the mean and SD of RMSEs from 200 realizations were plotted against the true R2* values with varying SNRs of 15, 30, 60. In clinical practice, the R2* mapping was firstly obtained by fitting the pixel-wise signals, then the R2* values in a homogeneous ROI were averaged to produce the representative R2* value for LIC quantification. For simulation, the averaged R2* value over the whole parenchyma than a ROI is more reasonable as a representative R2* value to reflect the overall level of LIC. However, in order to be consistent with *in vivo* data, a suitable ROI is selected and the R2* values over the ROI were averaged to produce a representative R2* value in the simulation. For each simulated R2* value and SNR, the mean and SD of representative R2* values from 200 repeats were calculated to evaluate the accuracy and precision of the ROI-based measurement using all three methods, *i.e*., fitting the original noisy data, the NLM-filtered and the ADSSW-filtered data with M^1^NCM model.

For the phantom study, 11 ROIs were manually delineated. Then, for each ROI, the coefficient of variation (COV) of the representative R2* values of 9 datasets with varying SNRs is calculated to assess the robustness of each method.

For the *in vivo* study, the histogram of SNRs (n = 128) with cumulative percentage was firstly plotted to illustrate the distribution of SNRs in this group data sets. Then, the representative R2* values of the 128 data sets were plotted to evaluate the performance of the ROI-based measurements among fitting the original noisy data, the NLM- and the ADSSW-filtered data with M^1^NCM model. In addition, R2* mappings of three subjects were presented for visual demonstration.

### The setting of the filtering parameters

In the NLM method, the search window and the patch size were respectively set to 11 × 11 and 5 × 5, which would produce a good performance for liver R2* measurement^29^. In the ADSSW method, the search window was also set to 11 × 11. In the simulation study, the parameter *h* was adjusted with an exhaustive search in a certain range (0.05 *σ* to 4 *σ*) to produce the minimum RMSE for both the NLM and our proposed algorithms; the parameter *σ* is the SD of simulated noise. In the phantom and clinical studies, the parameter *h* was set to 0.8 *σ* both in the NLM and ADSSW algorithms by visual inspection of the estimated R2* mapping; the parameter *σ* can be calculated as *σ* = $\sqrt{\mu /2L}$,where *μ* is the mean of the squared intensities in the background of the multi-echo images at all the TEs and *L* is the number of channels.

## Results

### Simulations Study

**Figure 1:**
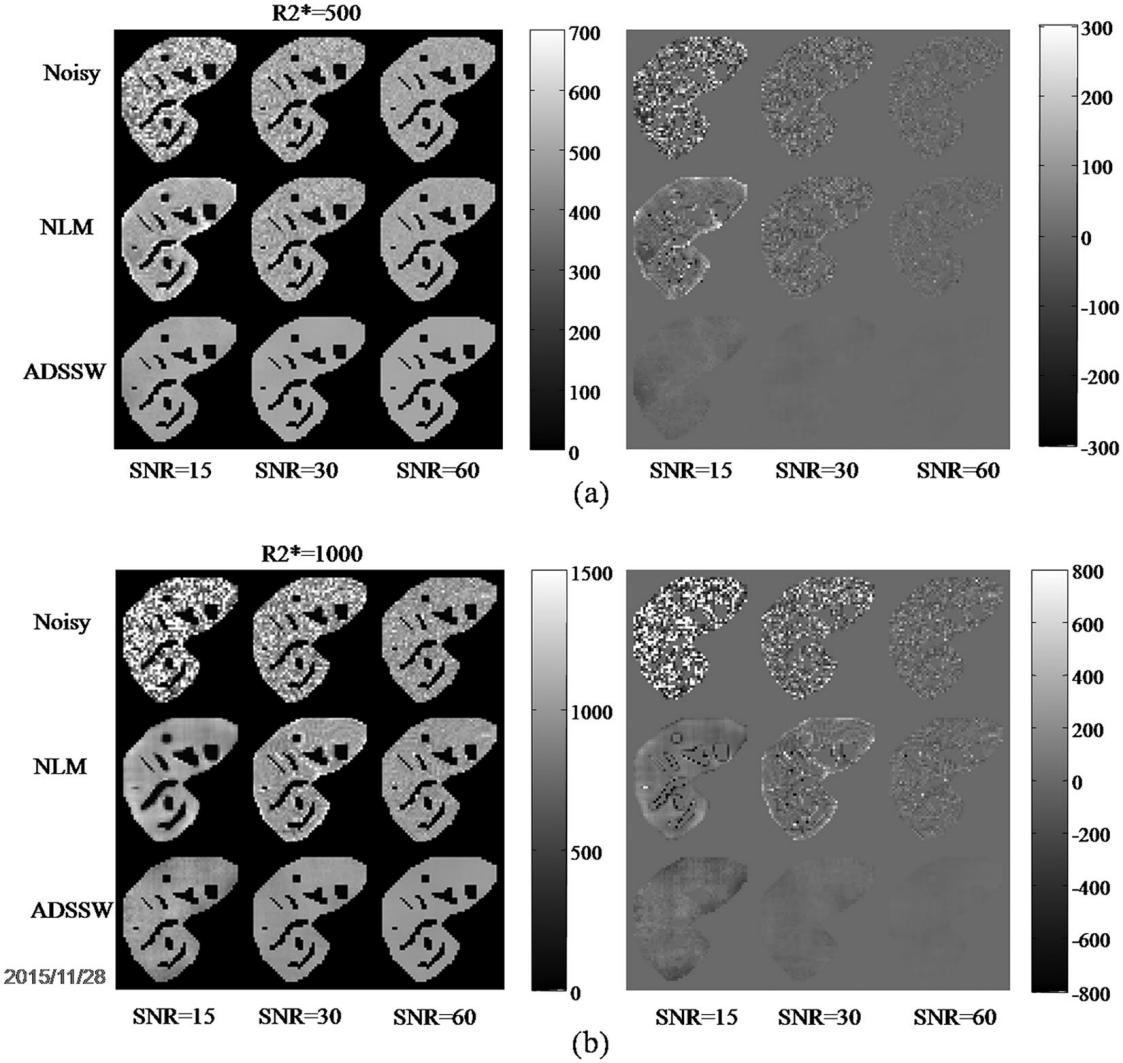
R2* mappings fitted from the original, NLM-, and ADSSW-filtered simulated data for SNR = 15, 30 and 60 with two true liver R2* values: (**a**) 500 s^−1^, (**b**) 1000 s^−1^. Left: the estimated R2* mappings. Right: the corresponding R2* error mappings.

Figure 1 presents the liver R2* mappings obtained from the noisy, NLM-filtered, ADSSW-filtered data and the corresponding error mappings with the true liver R2* of 500 s^−1^(a), 1000 s^−1^(b) and varying SNRs of 15, 30, 60. As shown in the left column of Fig. 1, both the NLM- and ADSSW-filtered data produced less noisy R2* mappings compared with those fitted from the noisy images. Blurred edges and a few outliers around vessels can be clearly observed in the R2* mapping fitted from the NLM-filtered images. The R2* mapping fitted from the ADSSW-filtered data has sharp edges between liver parenchyma and vessels without outliers around vessels. Those results can be more clearly observed from the corresponding error mappings shown in the right column of Fig. 1.

**Figure 2:**
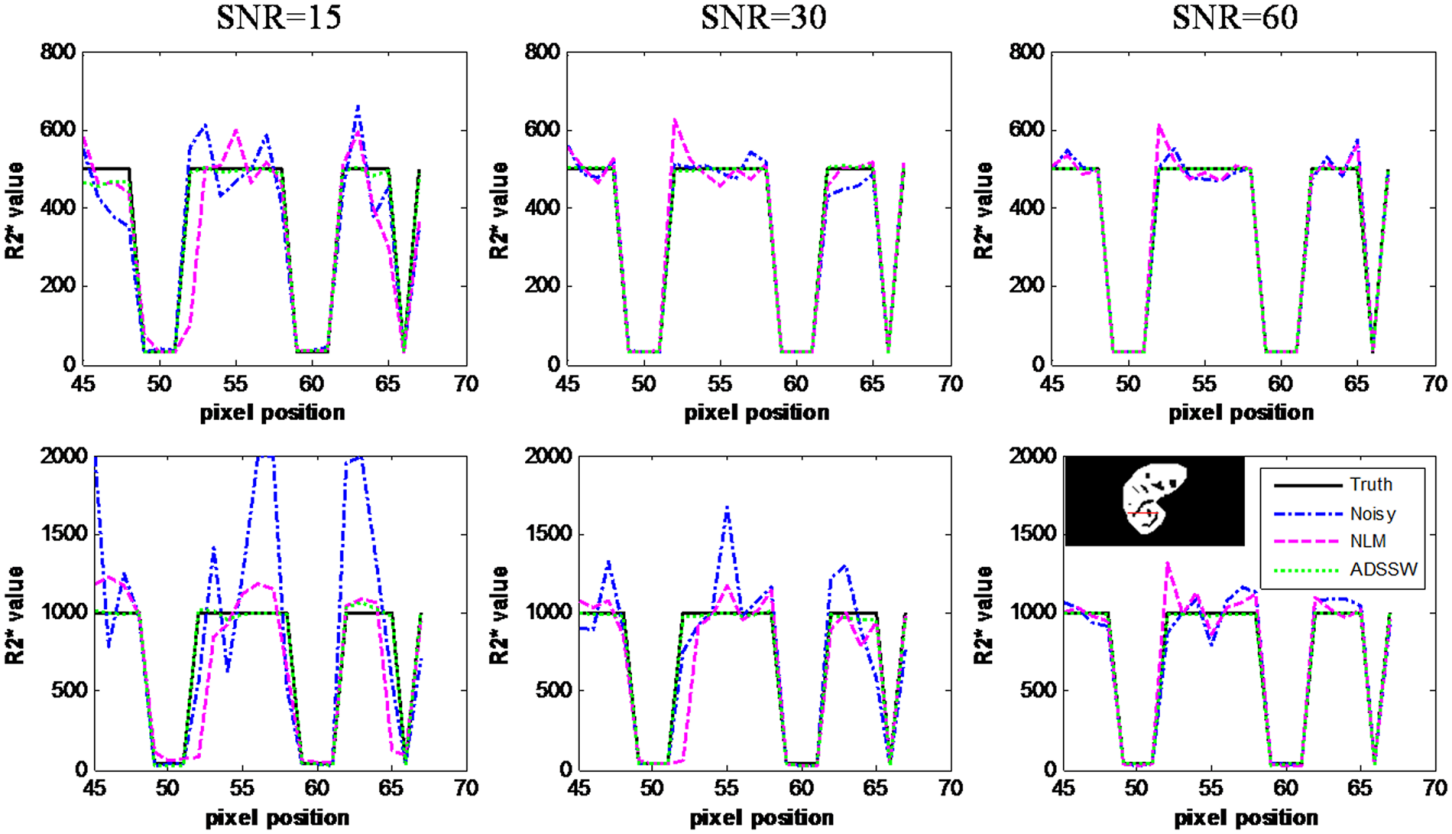
Plot of R2* values across a line in the R2* mapping fitted from NLM-, and ADSSW-filtered data, the true and the noisy R2* mapping in the simulation study. Top row: true R2* = 500 s^−1^. Bottom row: true R2* = 1000 s^−1^. Left to right: SNR = 15, 30 and 60.

To further illustrate the impact of filtering algorithms on the R2* measurement, the intensity values in the red line across the vessels were plotted as showed in Fig. 2. The estimated R2* values generated from the NLM-filtered serial images contain serious errors near the edges of rapid R2* variations. In contrast, the R2* values produced from the ADSSW-filtered images agree well with the true R2* values.

**Figure 3:**
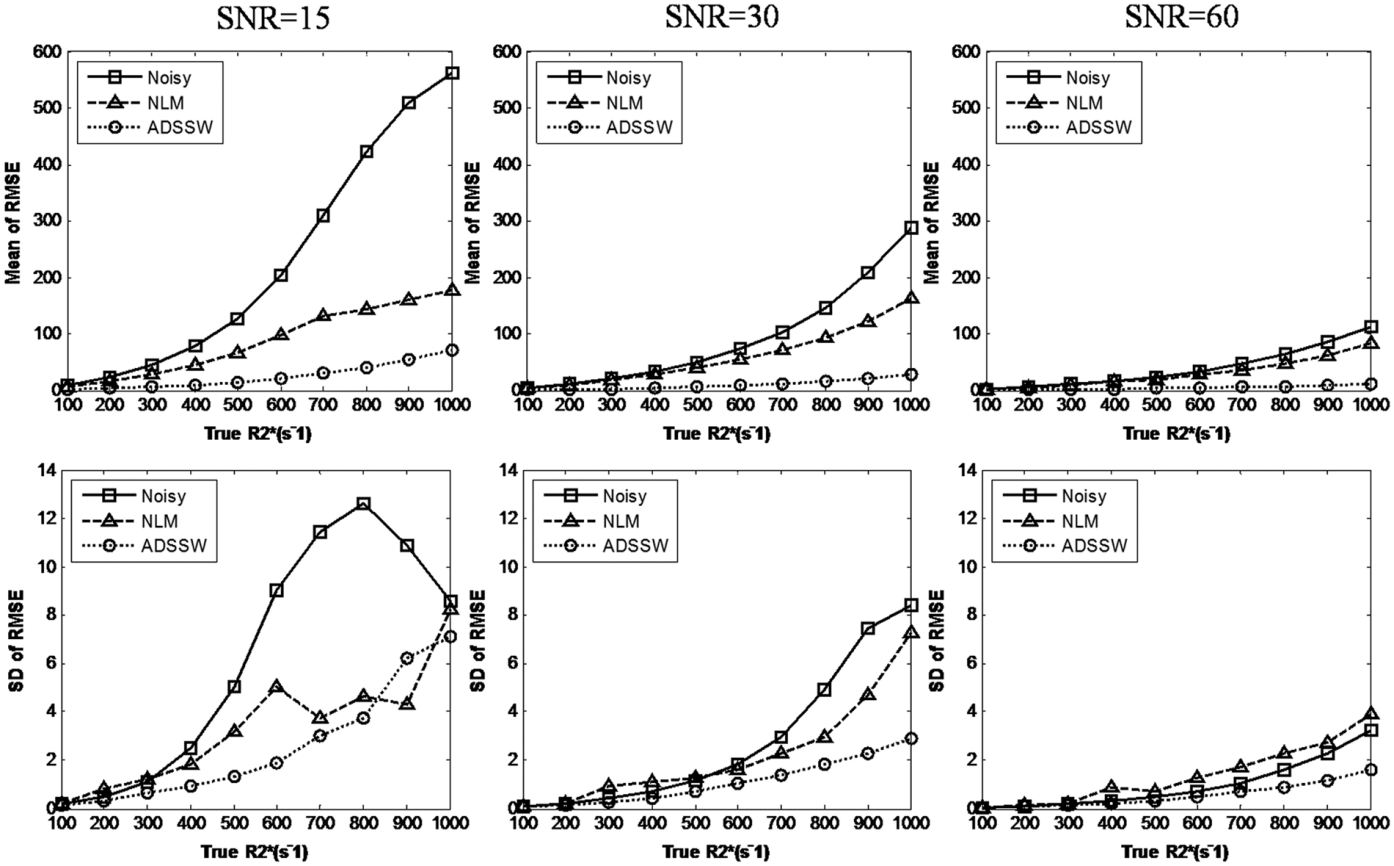
Plot of the means (top row) and SDs (bottom row) of the RMSEs of R2* mapping from 200 realizations against the true R2* values at varying noise levels (SNR = 15, 30 and 60).

The smoothing parameters *h* of the NLM and ADSSW algorithms are firstly optimized by the criterion of the RMSEs of R2* mappings. Then each R2* mapping was repeated 200 times with the optimized *h* at the same reference R2* value and SNR. Figure 3 shows the means and SDs of RMSEs of R2* mappings from 200 realizations for SNRs of 15, 30, 60, respectively. Obviously, the ADSSW-filtered data produced decreased RMSEs with smaller SDs than the noisy and NLM-filtered data.

**Figure 4:**
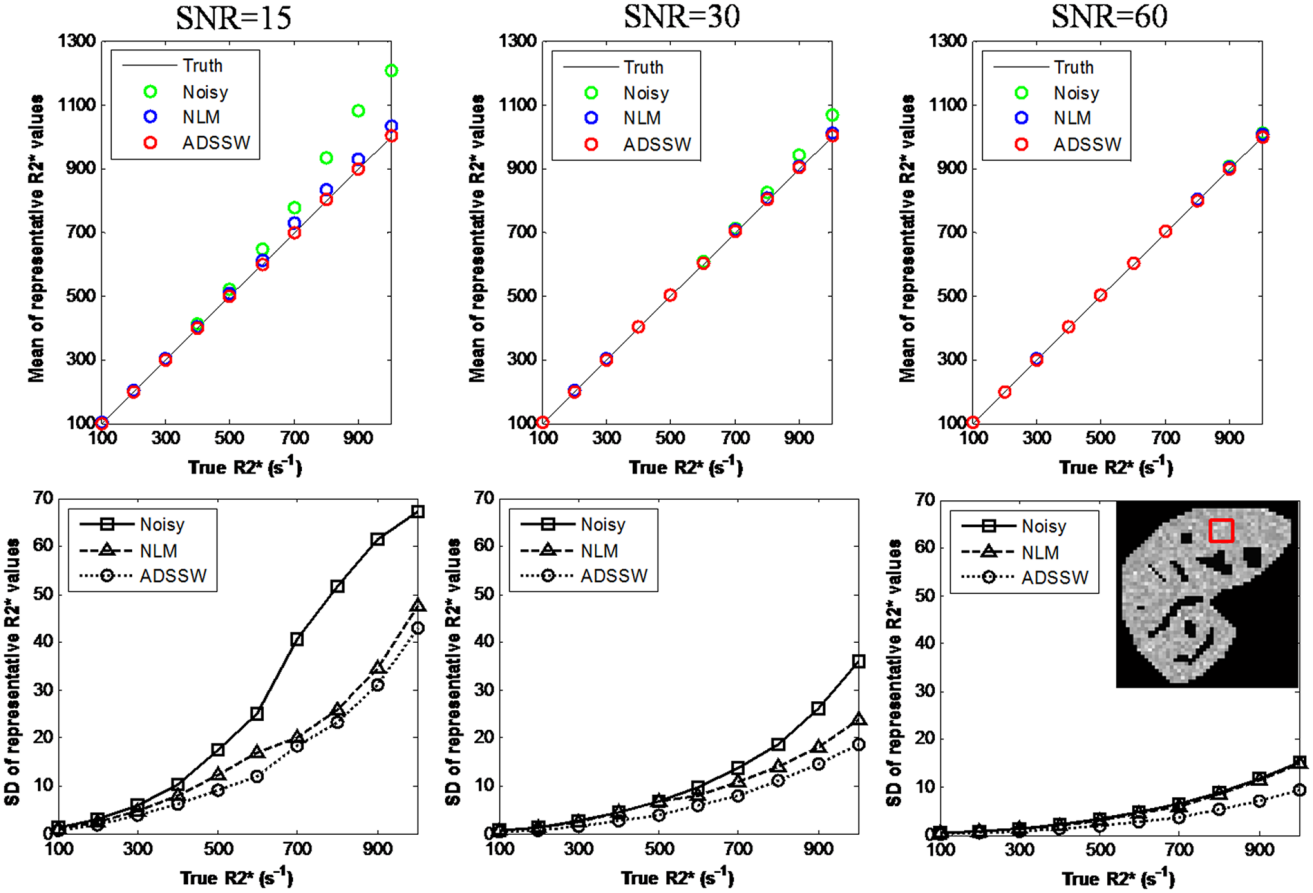
Plot of the means (top row) and SDs (bottom row) of representative R2* values from 200 repeats against the true R2* values at varying noise levels (SNR = 15, 30, 60).

To follow the clinical practice, the mean of the mapped R2* values in a ROI (8 × 8 pixels in the red box) was calculated as the representative R2* value. The mean and SD of the representative R2* values over 200 repeats were plotted against the varying true R2* values in Fig. 4. The first row of Fig. 4 shows that the noisy and NLM-filtered data produced overestimate of R2* value at low SNRs and high R2* values, while the ADSSW-filtered data produced more accurate R2* value without a bias. The second row of Fig. 4 shows that the SD of representative R2* values from ADSSW-filtered data was consistently lower than those from the noisy and NLM-filtered data which demonstrates that the ADSSW method is more robust against the Rician noise.

### Phantom Study

**Figure 5:**
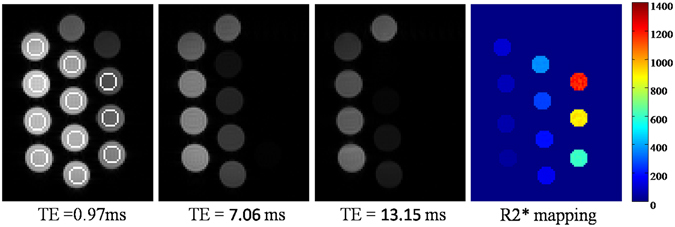
Example of the phantom images (AVG = 256) at TEs of 0.97 ms, 7.06 ms, 13.15 ms and the R2* mapping obtained from the less noisy images (AVG = 256). Circles in the first image indicate the selected ROIs.

Figure 5 shows the example images (AVG = 256) of the phantom at TE = 0.97, 7.06, 13.15 ms and the R2* mapping from the less noisy images with an AVG of 256. The circles in the most-left image indicates the selected ROIs for R2* measurements. Each ROI contained 69 pixels in this phantom study. The ROIs are respectively labelled as ROI1, ROI2, … ROI11 from top right to bottom left. Table 1 shows the COVs of the representative R2*s from 9 datasets with different SNRs for each selected ROI. ADSSW-filtered images produced smaller COVs of representative R2* values compared to the NLM-filtered images and corresponding no-filtered noisy images with varying SNRs (AVG = 1, 2, 4, 8, 16, 32, 64, 128 and 256). This again demonstrate that fitting the ADSSW-filtered images with M^1^NCM model is robust against the Rician noise.

**Table 1:** The COVs of the representative R2* values from 9 original noisy datasets with AVG = 1, 2, 4, 8, 16, 32, 64, 128 and 256, and their corresponding NLM- and ADSSW-filtered images.

	ROI 1	ROI 2	ROI 3	ROI 4	ROI 5	ROI 6	ROI 7	ROI 8	ROI 9	ROI 10	ROI 11
Noisy	0.0276	0.0142	0.0046	0.0050	0.0054	0.0026	0.0030	0.0045	0.0043	0.0053	0.0115
NLM	0.0181	0.0088	0.0048	0.0059	0.0071	0.0030	0.0038	0.0042	0.0056	0.0067	0.0118
ADSSW	**0.0123**	**0.0069**	**0.0045**	**0.0046**	**0.0034**	**0.0021**	**0.0025**	**0.0041**	**0.0037**	**0.0052**	**0.0110**

The minimum value in each column is highlighted in bold font.

### *In vivo* Study

**Figure 6:**
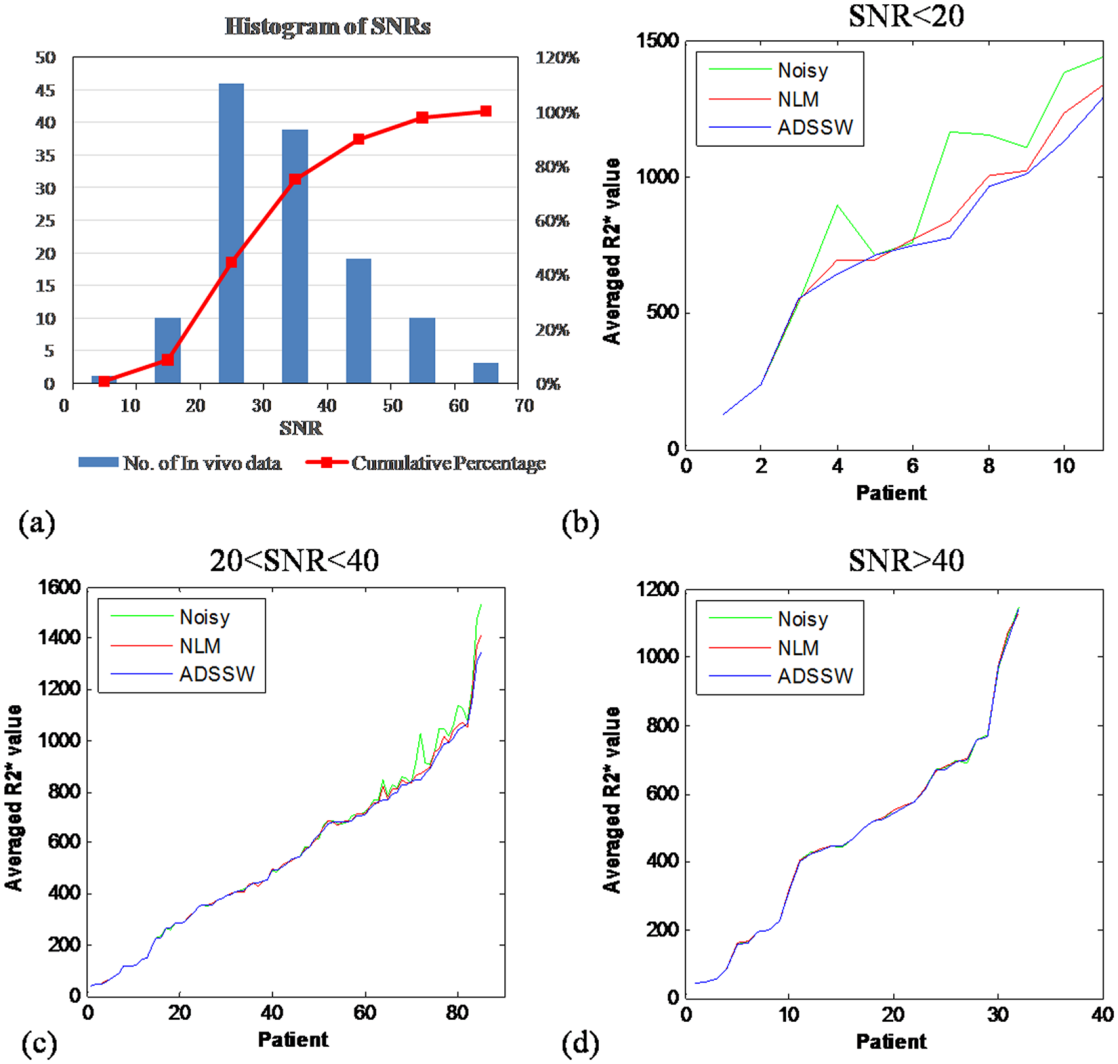
R2* quantification analysis for all the *in vivo* data sets (n = 128). (**a**): the histogram of the SNRs with cumulative percentage, (**b**–**d**): plot of the representative R2* values from the noisy, NLM- and ADSSW-filtered data in ascending order of R2* values for SNR < 20, 20 < SNR < 40 and SNR > 40, respectively.

Figure 6 shows the R2* analysis for all the subject data sets (n = 128). Figure 6a shows the histogram of the SNRs with cumulative percentage. The numbers of SNR < 20, 20 < SNR < 40 and SNR > 40 are respectively 11, 85 and 32 which corresponds to the figure (b), (c) and (d). In addition, the distribution of SNRs demonstrates that the SNRs of 15, 30, 60 in the simulation could well represent the poor, moderate and good SNRs of *in vivo* liver data. Figure 6b–d shows the results of the representative R2* values from the noisy, NLM- and ADSSW-filtered data in ascending order of R2* values from ADSSW-filtered data for SNR < 20, 20 < SNR < 40 and SNR > 40, respectively. At high R2* values with a low SNR, the representative R2* values from the noisy data are larger than those from the NLM- and ADSSW-filtered data; and those from NLM-filtered data are slightly larger than those from ADSSW-filtered data in most cases. These difference of R2* values among noisy, NLM- and ADSSW-filtered data become smaller as the SNR increases or the R2* value decreases. The R2* values from noisy, NLM- and ADSSW-filtered data are very close when the SNR > 40. These findings agree well with those of the simulation study.

**Figure 7:**
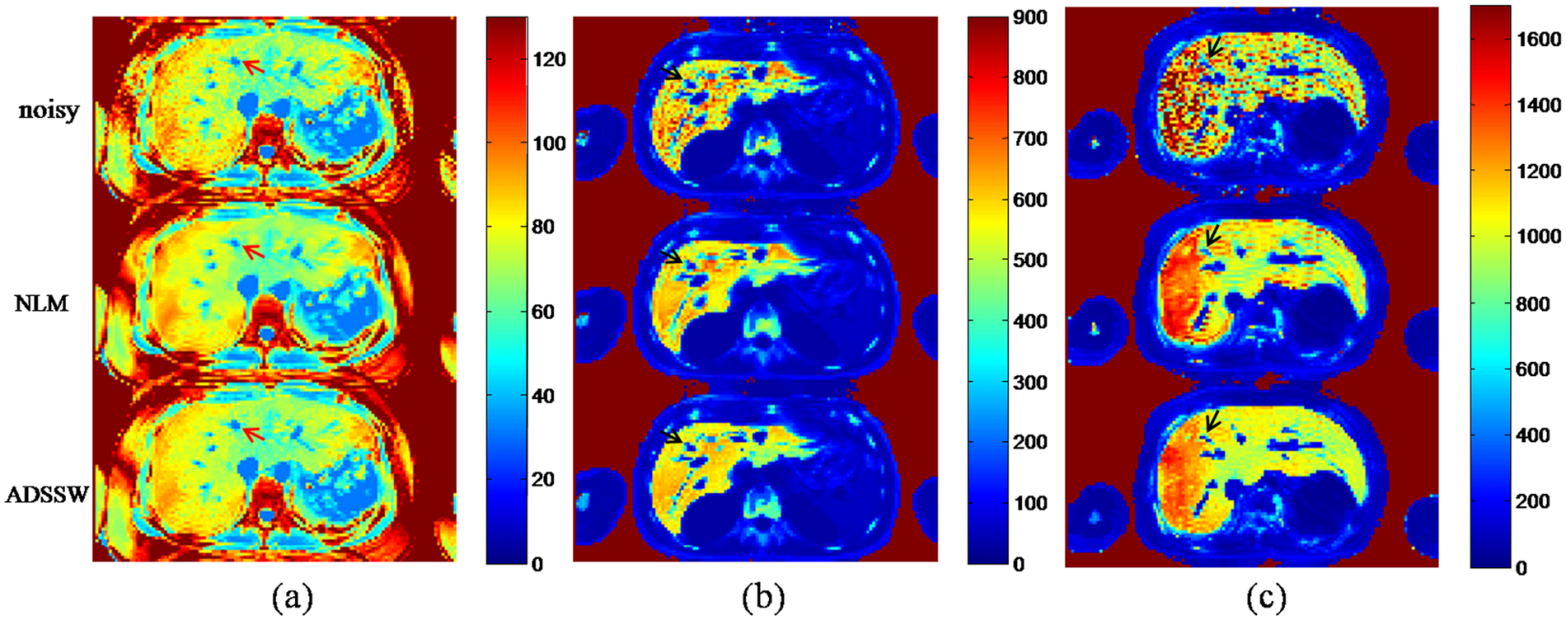
R2* mappings generated from the original, NLM-, and ADSSW-filtered *in vivo* liver images of three subjects with (**a**) normal, (**b**) moderate, and (**c**) severe LIC.

Figure 7 presents the R2* mappings of three subjects with normal (a), moderate (b), and severe(c) LIC generated from the original, NLM-filtered and ADSSW-filtered images. As seen in Fig. 7, fitting the NLM- and ADSSW-filtered data significantly reduce the noise of R2* mappings. In the R2* mapping estimated from the NLM-filtered images, some small vessels were slightly blurred (marked with arrows) and outliers existed near vessels and liver contour. In contrast, the shape of vessels were maintained well and no obvious outliers could be observed in the R2* mapping estimated from the ADSSW-filtered images. Visually, the values of R2* mapping obtained from ADSSW-filtered images seem smaller than the values from original and NLM-filtered images especially for serve LIC. This findings agree well with those from the simulation study which may be explained by that the original and NLM-based method overestimates the R2* values.

## Discussion

To reduce the noise influence on the R2* mapping, a novel scheme was proposed to filter the relaxometry images by averaging the decay curve before curve-fitting. The weight in the proposed method is calculated on the similarity between a neighbouring curve and the target curve in a local window which ensures that any pixel on the same decay curve have the same weight and hence avoids decay distortion introduced by using nonlinear algorithm such as the well-known NLM filter. In addition, the weight scheme calculated on two pixel-wise curves in our proposed algorithm is more reasonable than that in the NLM algorithm as the filter performance of the NLM algorithm will degrade if no sufficient similar patches are found which may often happens in a tiny structure such as a small vessel.

The simulated study demonstrated that the ADSSW algorithm outperforms the NLM algorithm in reducing variations in the homogeneous area and preserving the rapid-changing edges in both filtered images and estimated R2* mappings with varying noise levels. Quantitatively, the noisy and NLM-filtered data produced an overestimated representative R2* value at the low SNR and high reference R2* value while the ADSSW-filtered data produced an unbiased representative R2* value in all cases. For the *in vivo* study, the R2* mapping estimated from the ADSSW-filtered data is visually better with details preserved and no obvious outliers. The representative R2* values from the ADSSW-filtered data are slightly lower than those from noisy and NLM-filtered data at low SNR and high R2* values. These outcomes of the *in vivo* study are consistent with those of the simulation study.

The proposed ADSSW algorithm can be considered as a vector version of the Yaroslavsky filter which averages neighboring pixels with intensity-based similarity weights in a local search window^30^. The difference is that the ADSSW algorithm filters the decay signals as a vector by considering the similarity between two vectors instead of two pixels. The NLM filter^18^ determines average weights according to similarity between local patches and can be easily generalized into a vector form and applied to filter R2* relaxometry images to avoid any distortion to decay curves. In this scenario, the similarity-based weights are determined by the distance between local patches of decay signals. We have tried to filter R2* relaxometry images using the vector form NLM filter with different patch sizes (7 × 7, 5 × 5, 3 × 3), and found that their performance is inferior to that of the ADSSW algorithm in terms of the RMSE. This is probably because that it is difficult to find similar patches of signals, each of which consists of 12 intensity values along the TE dimension, especially near irregular edges. It should be noted that the ADSSW algorithm can also be regarded as a special case of the vector form NLM filter with patch size of 1, i.e., local patches are excluded from the weight determination.

For 12-echo *in vivo* liver images with a size of 64 × 128, the running time is about 400 s for all the three methods, *i.e*., the noisy, NLM and ADSSW methods including the M^1^NCM component. Note that the NLM- and ADSSW-filtered algorithms were implemented using C++ mex file which took only 4 s and 1 s respectively. The curve-fitting with M^1^NCM model, performed in Matlab code was the most time consuming part and can be speeded up using C++ implementation.

The performance of the ADSSW algorithm depends on the setting of the parameter *h* which controls the decay rate in transforming the Euclidean distances between decay signals to average weights. A large *h* corresponds to more smoothing effect and a small *h* means more rapid-changing details with less smooth effect. To make the proposed method robust, the knowledge of the noise (*σ*) is exploited to set the parameter, namely, *h* = *βσ*. In the phantom and *in vivo* study, parameter *β* were manually determined by checking the quality of filtered images and R2* mapping results which is one limitation of the current study. It is worth mentioning that the smoothing parameter of the NLM algorithm is also visually tuned in a same way to the proposed method. The automatic determination of optimal parameter *β* could be further investigated by combining the proposed method with objective metrics such as Stein’s unbiased risk estimate^31^.

In this study, the use of RMSE of R2* mapping as performance criterion has some drawbacks. Clearly, it favours techniques with reduced pixel-wise noise in R2* mappings. However, the clinical use of these R2* mappings is likely first for visual inspection (where nice maps with low noise are likely preferable), and then for ROI-based measurement over one or several regions that appear to have homogeneous R2* values. Thus, arguably what is clinically relevant is the performance of ROI measurements over a reasonably-sized ROI, rather than the per-pixel errors. In the scenario of the ROI-based measurements, the R2* bias becomes more of a concern rather than the noise of R2* mappings. Due to the stochastic property of noise, the representative R2* value calculated from the ROI with size of 8 × 8 is unstable and slightly varying for each realization in the simulation. For a more reliable result, the mean and SD of representative R2* values from 200 repeats were presented to evaluate the accuracy and precision of the ROI-based measurement. The ADSSW-filtered images produced unbiased representative R2* values with lower SDs in all cases while the noisy and NLM-filtered images produced biased representative R2* values at high reference R2* values with low SNRs.

Owing to the hardware limit, a relatively long first TE of 1 ms is often used for clinical patient scans. This is the reason we used a similar long first TE in our simulation and phantom studies. However, the signal rapidly decays to a plateau at early TEs for high R2* values. In this scenario, an ultrashort TE (UTE) sequence^32, 33^ will improve the detection of early signals and may reduce bias and variance of the R2* mapping contaminated by the noise and severe iron overload. However, the UTE sequence is not routinely used due to its complexity. A future study to explore the feasibility and benefits of the UTE sequence in tissue iron quantification is guaranteed. By contrast, a protocol including long late echo times (up to 16 ms in our study) is often used in patient scans to cover a wide range of R2* values from patients with severe iron overload and normal conditions. In practice, it is hard to predict the liver R2* value before the patient scan. Hence it can pose a challenge for the operator to determine optimal TEs to use. The operator can choose to repeat the scan with an optimized protocol, but obviously at extra scan time and cost. Again, there is a need of future studies to determine how TEs can affect the R2* mapping and whether there is an improved protocol to guide patient scans.

It should be noted that the fat saturation technique was employed in this study. The presence of liver fat will result in protocol-dependent bias in R2* measurement when using the M^1^NCM model which accounts for only the noise-related bias but not the fat-related bias. However, the ADSSW filtering method is independent of the fitting model and thus can be extended to the fatted case by fitting the ADSSW-filtered data with the fat-corrected fitting model^34^.

Another limitation is that the current study assumes uniform noise distribution across the whole image. This assumption generally holds when no subsampling is employed for acceleration and no significant correlation exists among array coils. Parallel magnetic resonance imaging techniques, such as sensitivity encoding (SENSE)^35^ and generalized auto calibrating partially parallel acquisitions (GRAPPA)^36^, introduce spatially varying noise levels across the image. In such scenarios, the ADSSW algorithm can be adapted to the spatially varying noise using the local noise level *σ*_*ij*_ to adjust the amount of denoising strength where *σ*_*ij*_ can be obtained from the image using a local noise estimation^37^. We plan to extend the proposed ADSSW algorithm to handle the non-uniform noise in the future.

In conclusion, a novel method of averaging decay curves in a local window with similarity-based weights was presented. The simulation and experimental results demonstrate that the proposed method outperforms the conventional NLM and produces accurate R2* mapping for improved LIC quantification. More patient studies are needed to confirm the clinical practicality of the proposed method.
